# Selective Adsorption of Pb(II) from Aqueous Solution by Triethylenetetramine-Grafted Polyacrylamide/Vermiculite

**DOI:** 10.3390/ma11040514

**Published:** 2018-03-28

**Authors:** Shiqing Gu, Lan Wang, Xinyou Mao, Liping Yang, Chuanyi Wang

**Affiliations:** 1 Laboratory of Environmental Sciences and Technology, Xinjiang Technical Institute of Physics & Chemistry, Key Laboratory of Functional Materials and Devices for Special Environments, Chinese Academy of Sciences, Urumqi 830011, China; gusq@ms.xjb.ac.cn (S.G.); maoxy@ms.xjb.ac.cn (X.M.); Yanglp@ms.xjb.ac.cn (L.Y.); 2 University of Chinese Academy of Sciences, Beijing 100049, China

**Keywords:** amine groups, amino-functionalized, vermiculite, adsorption, Pb(II) removal

## Abstract

Amine groups play significant roles in polymeric composites for heavy metals removal. However, generating a composite with a large number of functional and stable amine groups based on clay is still a challenge. In this work, a new amine-functionalized adsorbent based on acid-activated vermiculite (a-Verm) was prepared by organic modification of silane coupling agent as bridge, followed by in situ polymerization of acrylamide (AM) and further grafting of triethylene tetramine (TETA). The obtained polymeric composite g-PAM/OVerm was characterized by scanning electron microscope (SEM), energy dispersive spectrometer (EDS), Fourier transform infrared (FTIR), thermal analysis (TG/DTG), X-ray photoelectron spectroscopy (XPS) and Brunauer–Emmett–Teller (BET) analyses, confirming that amine groups were successfully grafted onto the surface of Verm. The efficacy g-PAM/OVerm for removing Pb(II) was tested. The adsorption equilibrium data on g-PAM/OVerm was in good accordance with the Langmuir adsorption isotherms, and the adsorption maximal value of Pb(II) was 219.4 mg·g^−1^. The adsorption kinetic data fit the pseudo-second-order kinetic model well. Additionally, g-PAM/OVerm has better selectivity for Pb(II) ion in comparison with Zn(II), Cd(II) and Cu(II) ions. The present work shows that g-PAM/OVerm holds great potential for removing Pb(II) from wastewater, and provides a new and efficient method for the removal of heavy metal ions from industrial wastewater.

## 1. Introduction

In recent years, heavy metal pollution is becoming more and more serious due to the rapid development of industrial activities such as mining and metallurgy [1,2]. Among the most hazardous heavy metal ions, Pb(II) has attracted much attention due to its strong and persistent toxicity even at low concentrations [3,4,5]. Consequently, the demand for effective and economical removal methods of lead compounds from aquatic environment is growing. Removal of Pb(II) is generally carried out via membrane separation [6], chemical precipitation [7], solvent extraction [8] ion-exchange [9], and adsorption [10,11,12]. Among these techniques, adsorption has become more popular in recent years, due to its availability and lower cost in industrial applications [13,14].

Natural clay materials are capable of removing heavy metals from aquatic environments, due to their chemical and mechanical stability compared with other adsorbents [15,16]. In particular, vermiculite has become one of the most studied clay materials. As an important part of layered silicate, vermiculite has a 2:1 alum inosilicate layered structure. The 2:1 layer consists of an tetrahedral sheet of silica and two sheets of octahedral MgO_2_(OH)_4_ [17,18]. Exchangeable cations located in the interlayer space, such as K^+^, Na^+^, Ca^2+^ and Mg^2+^, compensate for the positive charge deficiency that exists in the parallel 2:1 layers [19,20]. However, these materials actually possess poor adsorption capacity for heavy metal ions on the surface, since adsorption occurs mainly by ion exchange process in the interlayer.

Many reports have shown that inorganic or organic modified vermiculite has relatively greater adsorption capacity for heavy metal ions than raw vermiculite, because these additives provide more active sites or stronger binding with heavy metals [21,22,23]. In addition, acid treatment can increase the specific surface area of vermiculite and remove mineral impurities by partially dissolving the external layers, leading to formation of additional silicon hydroxyl (Si–OH) without destroying the original layered structure [24,25]. The reactivity of the Si–OH groups makes chemical modifications on the surface of vermiculite easy to accomplish, and these modifications can strengthen the vermiculite affinity for different compounds [26]. Moreover, the chemical modification of the vermiculite surface with organic reagents, including ammonium citrate tribasic [27], 2,2-bis(hydroxymethyl)-propionic acid [28], *N*-methylimidazole [29], octodecyl trimethyl ammonium chloride [30] and polyacrylamide [31,32,33], can be applied to enhance the adsorption capacity for heavy metal ions [34]. For these organic species, amine groups provide the major binding sites for heavy metal adsorption. Polymers like polyacrylamide can be deposited onto the surface of clay with the help of the functional bridging reaction between silane coupling agent and Si–OH groups in the surface of clay [35,36,37]. Additionally, as the further modification, the Mannich grafting reaction is adopted to graft triethylenetetramine onto the chain of polyacrylamide, making the composite contain large number of amine groups which can enhance the adsorption capacity for heavy metal ions. Furthermore, the combination of organic polymer and clay can not only increase the adsorption capacity for heavy metal ions, but also improve the intensity and stability of the adsorbent depending on the natural property of clay; at the same time, acid-treated clay with a large specific surface area can provide a proper matrix for the efficient deposition of polymers [38,39,40]. In this research, polyacrylamide with a large number of amide groups was grafted onto the surfaces of vermiculite clay. In addition, the Mannich grafting reaction was adopted to greatly increase the number of –NH_2_ binding sites. However, successful grafting of the polyacrylamide (PAM) macromolecular chain onto the surface chemically modified vermiculite (Verm) minerals, with large numbers of –NH_2_ groups, is still scarcely reported.

The purpose of this study was to prepare the triethylenetetramine-grafted polyacrylamide/vermiculite (g-PAM/OVerm) adsorbent with a large number of amine functional groups based on in situ polymerization of acrylamide and subsequent graft polymerization of triethylenetetramine onto the surfaces of PAM/OVerm via Mannich reaction. Vermiculite was activated with hydrochloric acid to obtain a greater specific surface area and more silanol groups. Then, the activated vermiculite was organically modified by silanization using 3-(triethoxysilyl)propyl methacrylate, in which silane bridges provided more stable bonds with the polymer. The obtained materials were tested for their ability to remove Pb(II) through equilibrium and kinetic sorption experiments. The effects of the graft polymerization reaction conditions and the pH of the aqueous solution on the adsorption process were evaluated.

## 2. Materials and Methods

### 2.1. Materials and Reagents

The raw vermiculite was supplied by Xinjiang Yuli Xinlong vermiculite Co. Ltd. (Korla, China), with an average size of 7 mm. Acrylamide (AM), 3-(triethoxysilyl)propyl methacrylate (TEPM) and triethylenetetramine (TETA) were purchased from Shanghai Chemical Reagent Co. Ltd. (Shanghai, China) and 2,2-Azobisisobutyronitrile (AIBN) was purchased from Chengdu Kelong Chemical Reagents Ltd. (Chengdu, China). Toluene, formaldehyde solution, hydrochloric acid and anhydrous ethanol were of analytical grade and purchased from Sigma-Aldrich (St. Louis, MO, USA). Milli-Q water (18 MΩ·cm) was used for the preparation of all solutions.

### 2.2. Modification of Verm

The procedure to modify Verm follows the protocol as described in previous work [19,41,42]. The raw vermiculite (Verm) was acid-treated as follows: 2 g vermiculite was dispersed in a 2 M HCl solution and stirred for 12 h. The mixture was filtered and washed thoroughly with water until the filtrate reached a neutral pH. The obtained material was dried at 110 °C for 8 h and denoted as acid-activated Verm (a-Verm). In a next step, the a-Verm was organically modified as follows: 3.0 g Verm, 1.0 mL water and 3.0 mL TEPM were added to 100 mL toluene with ultrasonic agitation for 30 min. The mixture was then refluxed with electromagnetic stirring at 45 °C for 4 h. The solid was separated by centrifugation and washed with toluene, anhydrous ethanol and water to remove excess modifier and possible products of hydrolysis. The obtained sample was dried in a desiccator at 80 °C. The final 3-(triethoxysilyl)propyl methacrylate-modified acid vermiculites were abbreviated as OVerm.

### 2.3. Preparation of PAM/OVerm

The procedure to prepare PAM/OVerm follows the steps of previous work [33,43]. OVerm (2.0 g) was dispersed in a 250-mL three-neck flask with 100 mL of toluene solution and stirred for 10 min. To the mixture, 0.6 g AM monomer and 0.06 g AIBN initiator were added. The mixture was refluxed with stirring at 80 °C for 6 h in an oil bath under N_2_ atmosphere. Finally, the resulting sample was separated by centrifugation, washed with toluene and anhydrous ethanol, and dried at 80 °C. The obtained sample was denoted as PAM/OVerm.

### 2.4. Preparation of g-PAM/OVerm

**Scheme 1:**
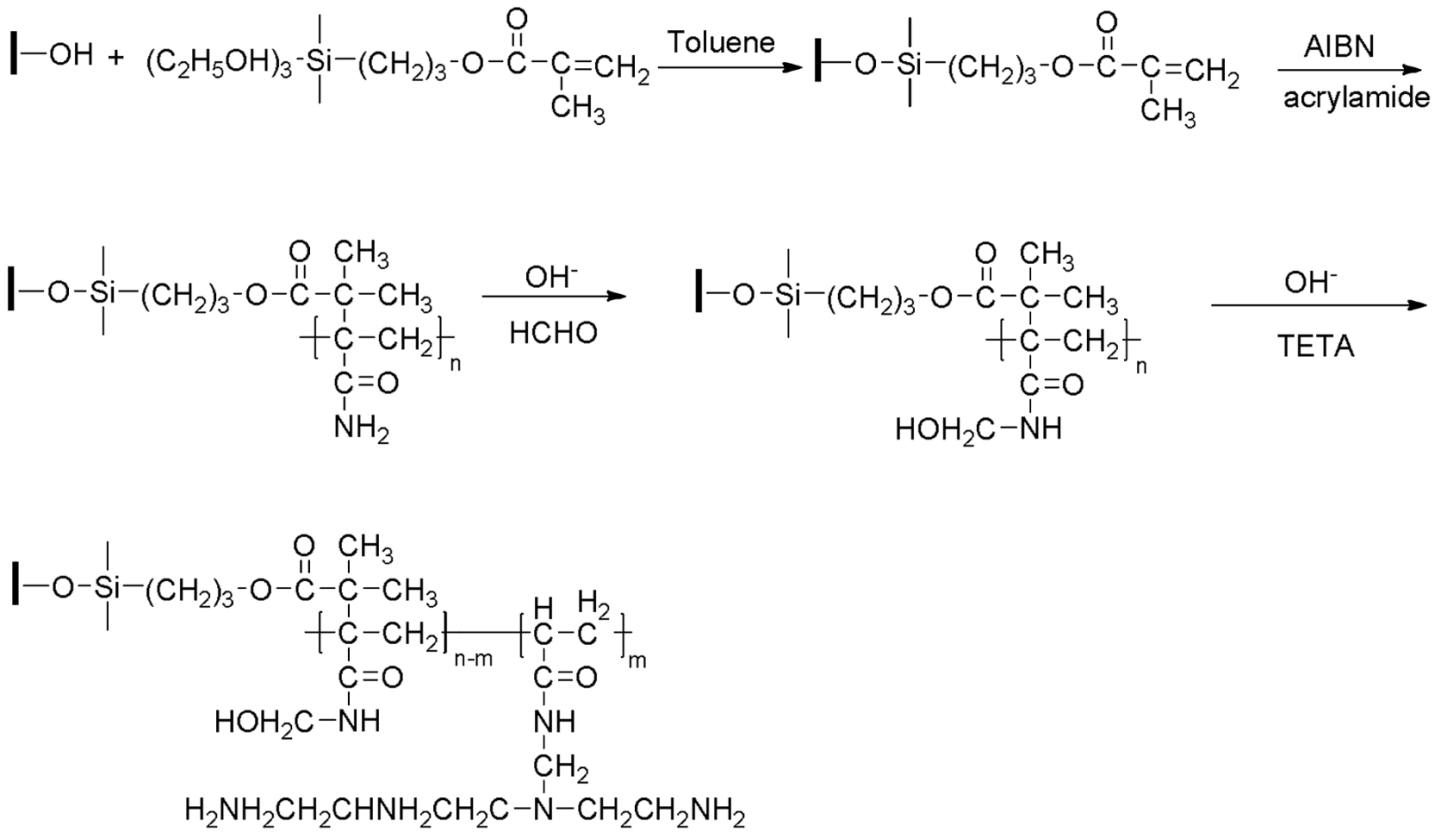
The preparation of triethylenetetramine-grafted polyacrylamide/vermiculite (g-PAM/OVerm).

The procedure to prepare g-PAM/OVerm follows the steps of previous work [44]. Triethylenetetramine (TETA) was incorporated to the PAM/OVerm to introduce more –NH_2_ groups onto the long chain of PAM. PAM/OVerm (1.0 g) was dispersed in a 250 mL flask containing 99 mL distilled water. The pH of the mixture was adjusted to a specified value with sodium hydroxide or hydrochloric acid solution. The mixture was stirred at a specified temperature, and 1.3 mL formaldehyde was added. After a specified time, 1.5 mL TETA was added dropwise, and the reaction continued for a period of time. Finally, the grafted PAM/OVerm was separated by centrifugation and washed with distilled water until the pH of the wash was near neutral. The product was dried at 80 °C. The obtained adsorbent materials were abbreviated as g-PAM/OVerm. A schematic of the preparation of g-PAM/Overm is presented in Scheme 1.

### 2.5. Characterization

Fourier transform infrared spectroscopy (FTIR) measurements were conducted on a Nicolet is 50 instrument (Thermo Fisher Scientific, Waltham, MA, USA) at a resolution of 4 cm^−1^ in the region of 400–4000 cm^−1^ using KBr for dilution. The morphology of the sample was observed with field-emission scanning electron microscopy (FE-SEM, Zeiss Supra55VP, Oberkochen, Germany) operated at an accelerating voltage of 20 kV, after coating the samples with gold film. Thermal analysis (TG and DTG) was performed with a Netzsch STA449F3 instrument system (NETZSCH Instrument Co. Ltd., Selb, Germany) at a scan rate of 10 °C·min^−1^ up to 800 °C in N_2_ atmosphere. N_2_ isotherms were obtained at −196 °C using a surface area and pore size analyzer (NOVA 2200e, Quantachrome, Boynton Beach, FL, USA). The specific surface areas (S_BET_) were calculated using the Brunauer–Emmett–Teller (BET) equation within a relative pressure range (P/P_0_) of 0.05–0.30. The pore volume was determined from the amount of N_2_ adsorbed at the highest relative pressure of P/P_0_ ~ 0.99. XPS analyses were performed using an ESCALAB 250 spectrophotometer (Thermo Fisher Scientific, Waltham, MA, USA) with an Al KαX-ray source (1486.6 eV of photons).

### 2.6. Adsorption Experiments

Adsorption experiments for Pb(II) were carried out to determine the optimum conditions for chemical modification. Pb(NO_3_)_2_ was used to prepare the Pb(II) solutions. Pb(II) adsorption was carried out in a 250 mL iodine flask by dispersing 50 mg of the adsorbent in 25 mL Pb(II) aqueous solutions (400 mg·L^−1^) at different pH values. The mixtures were shaken via a constant temperature vibration shaker at 150 rpm and 30 °C. The pH of solutions was adjusted by sodium hydroxide solution or dilute nitric acid and measured by a pH meter (Mettler Toledo 320). The adsorbent was separated by centrifugation, and the Pb(II) concentration was measured by ICP-OES (VISTA-PRO CCD Simultaneous, Mason, OH, USA). The adsorption isotherms were measured in the range of 50–800 ppm at 30 °C. The effects of contact time on Pb(II) adsorption and kinetics were studied in the range of 2–120 min.

The effects of Pb(II) adsorption by g-PAM/OVerm with variable pH, temperature and reaction time during the grafting Mannich reaction was studied to obtain the optimal conditions for chemical modification. The adsorbed amount of Pb(II) at equilibrium was calculated according to the difference in Pb(II) concentration before and after adsorption, based on the following equation:

(1) $$q=\frac{(C_{i}-C_{f})V}{m},$$

where q is the Pb(II) uptake capacity by the adsorbent (mg·g^−1^), *C*_i_ is the concentration of Pb(II) in solution before adsorption (mg·L^−1^), *C*_f_ is the concentration of Pb(II) in solution after adsorption (mg·L^−1^), m is the dry weight of the adsorbent (g), and *V* is the volume of Pb(II) solution (L).

### 2.7. Regeneration of the Adsorbent

After the adsorption test, the adsorbent was desorbed by placing it in 25 mL dilute hydrochloric acid (1 M) and shaking at 60 °C for 60 min. After desorption, the adsorbent and liquid phase were separated by centrifugation at 6000 rpm for 10 min. The initial and final Pb(II) concentrations were measured by ICP-OES. Continuous adsorption–desorption experiments were carried out for five cycles to determine the reusability of the g-PAM/OVerm.

## 3. Results and Discussion

### 3.1. The Effects of the Grafting Reaction Conditions on Adsorption

#### 3.1.1. pH of Solution

**Figure 1:**
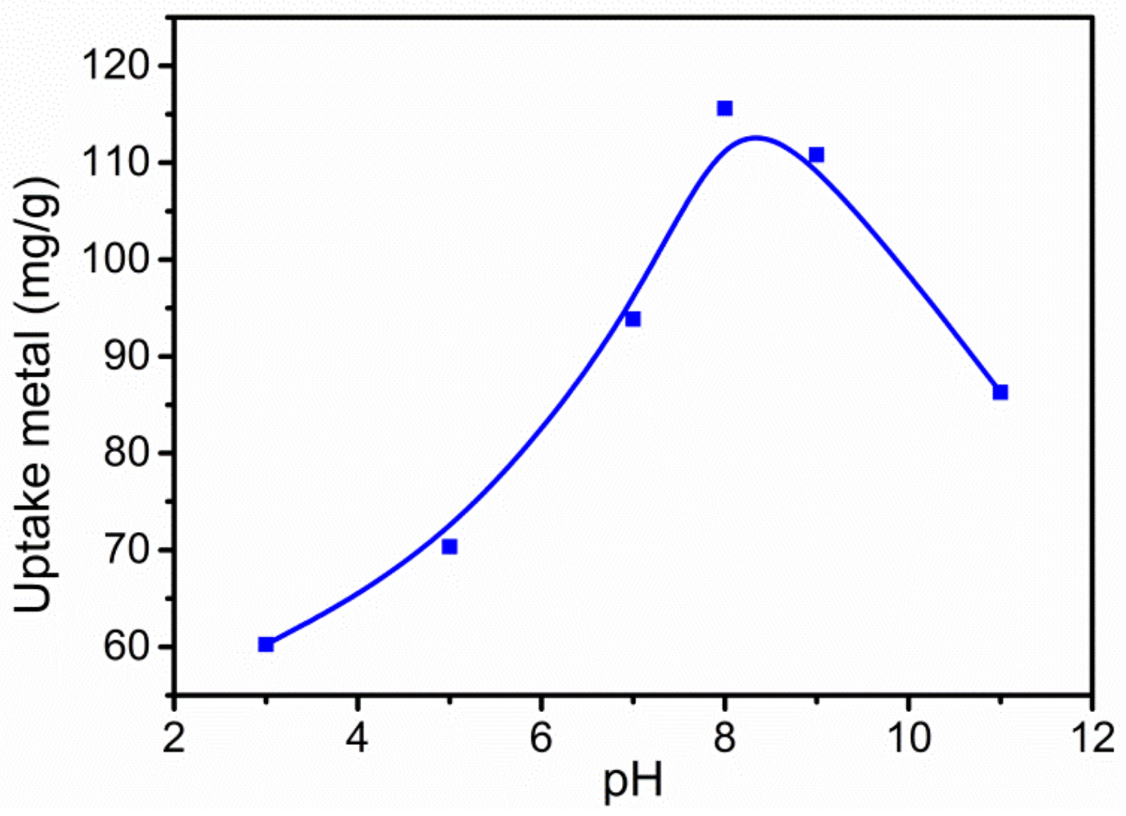
Effect of the pH of the grafting reaction on Pb(II) removal and q_e_. (Reaction conditions: *C*_i_ = 400 mg·L^−1^, T = 50 °C, grafting reaction times t_1_ = 1 h, t_2_ = 1 h)

Figure 1 shows the effect of the initial pH of the grafting reaction solution on the adsorption capacity of g-PAM/OVerm for Pb(II). It can be seen that the Pb(II) adsorption capacity increased significantly as the pH increased, reaching a maximum value of 115.6 mg·g^−1^ at pH 8. When the pH value continued to increase, the adsorption capacity decreased, indicating that a suitable alkaline environment is conducive to obtain a higher grafting rate of –NH_2_ groups on g-PAM/OVerm. Thus, the optimal pH of the grafting reaction was 8, and this condition was used for the rest of the batch experiments.

#### 3.1.2. Temperature of the Grafting Reaction

**Figure 2:**
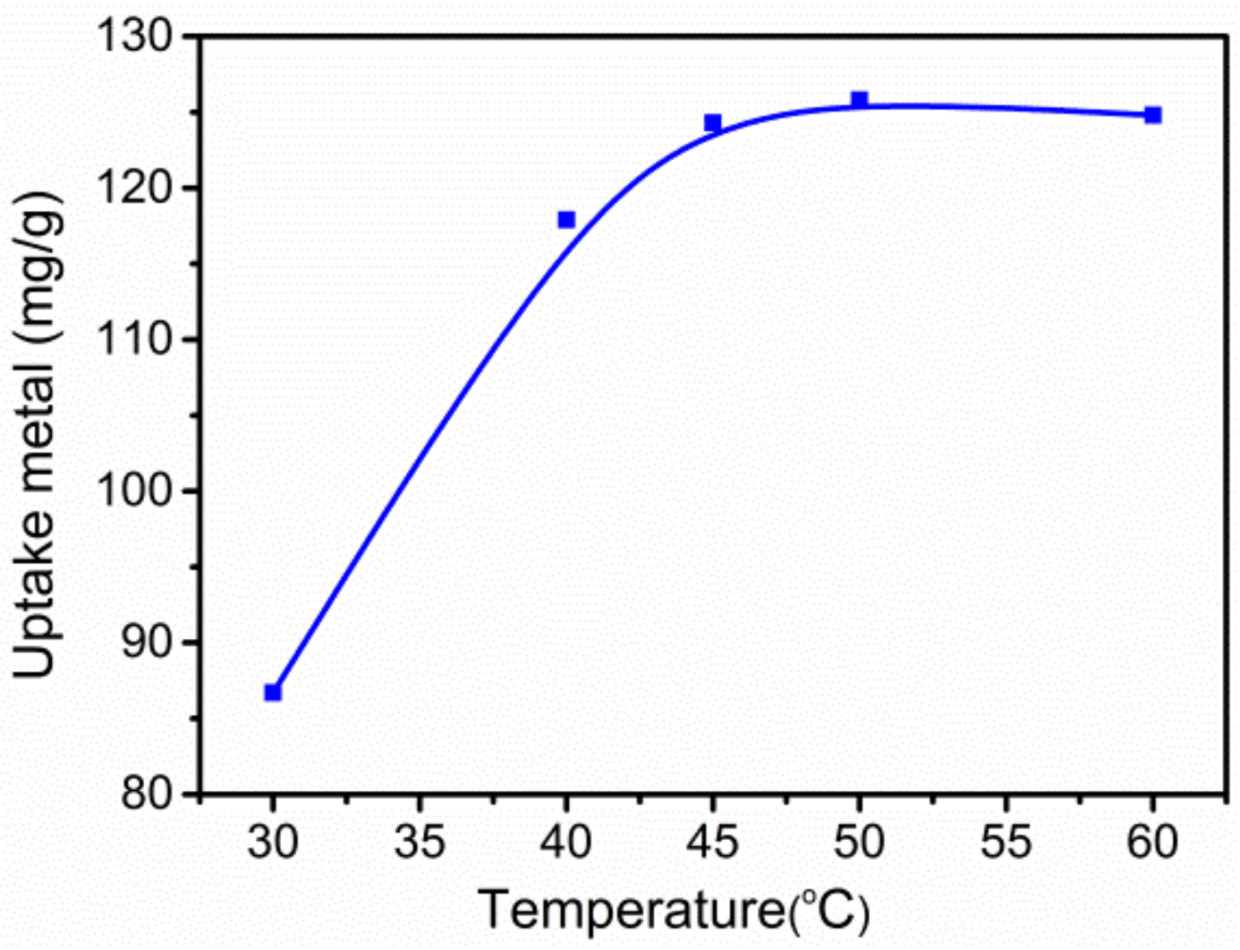
Effect of temperature of the grafting reaction on Pb(II) removal and q_e_. (Reaction conditions: *C*_i_ = 400 mg·L^−1^, pH = 8, grafting reaction times t_1_ = 1 h, t_2_ = 1 h.)

Figure 2 shows the effect of the grafting reaction temperature on Pb(II) adsorption. Clearly, adsorption capacity for Pb(II) on g-PAM/OVerm increased with increasing reaction temperature, reaching a maximum of 125.8 mg·g^−1^ at 50 °C and then remaining constant. This is because higher temperature promotes molecular locomotion and diffusion velocity of PAM molecular chains, formaldehyde and TETA molecules, resulting in more effective NH_2_-grafting. The system temperature of the grafting reaction at 50 °C was used for the rest of the batch experiments.

#### 3.1.3. Duration of the Grafting Reaction

**Figure 3:**
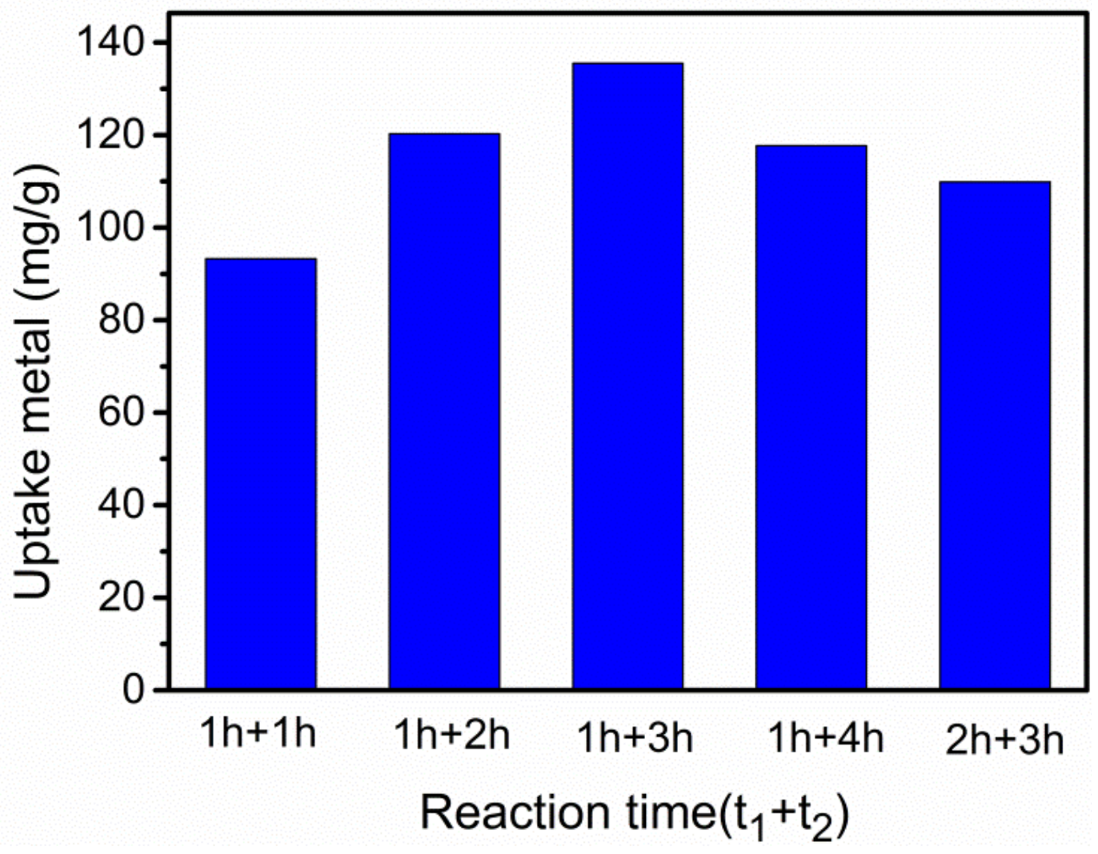
Effect of the grafting reaction times on Pb(II) removal and q_e_. (Reaction conditions: *C*_i_ = 400 mg·L^−1^, pH = 8, T = 50 °C.)

According to the previous steps, the pH of the reaction system was immobilized as 8, the grafting temperature was determined as 50 °C simultaneously. The duration of the grafting reaction was optimized. For the two-step reaction of –NH_2_ grafting reaction, t_1_ was the time at which the hydroxymethyl derivative appeared, and t_2_ was the reaction time of the hydroxymethyl derivative and TETA. These two parameters were altered in different combinations. Figure 3 shows that the optimal reaction times (t_1_ and t_2_) for NH_2_-grafting were one hour and three hours, respectively. Increasing t_2_ could improve Pb(II) removal capacity with g-PAM/OVerm within a certain range. The reaction time t_1_ of one hours was sufficient for hydroxylation; thus, the grafting reaction times of one hour and three hours for t_1_ and t_2_, respectively, were selected for the following experiments.

According to the adsorption tests above, the optimal grafting conditions were pH at 8, grafting reaction temperature at 50 °C and the combination of grafting reaction time as 1 h and 3 h. Similar work involve pH, temperature and duration of the reaction was studied by Yijiang Zhao and Shouyong Zhou [31,43].

### 3.2. Characterization

#### 3.2.1. FTIR

**Figure 4:**
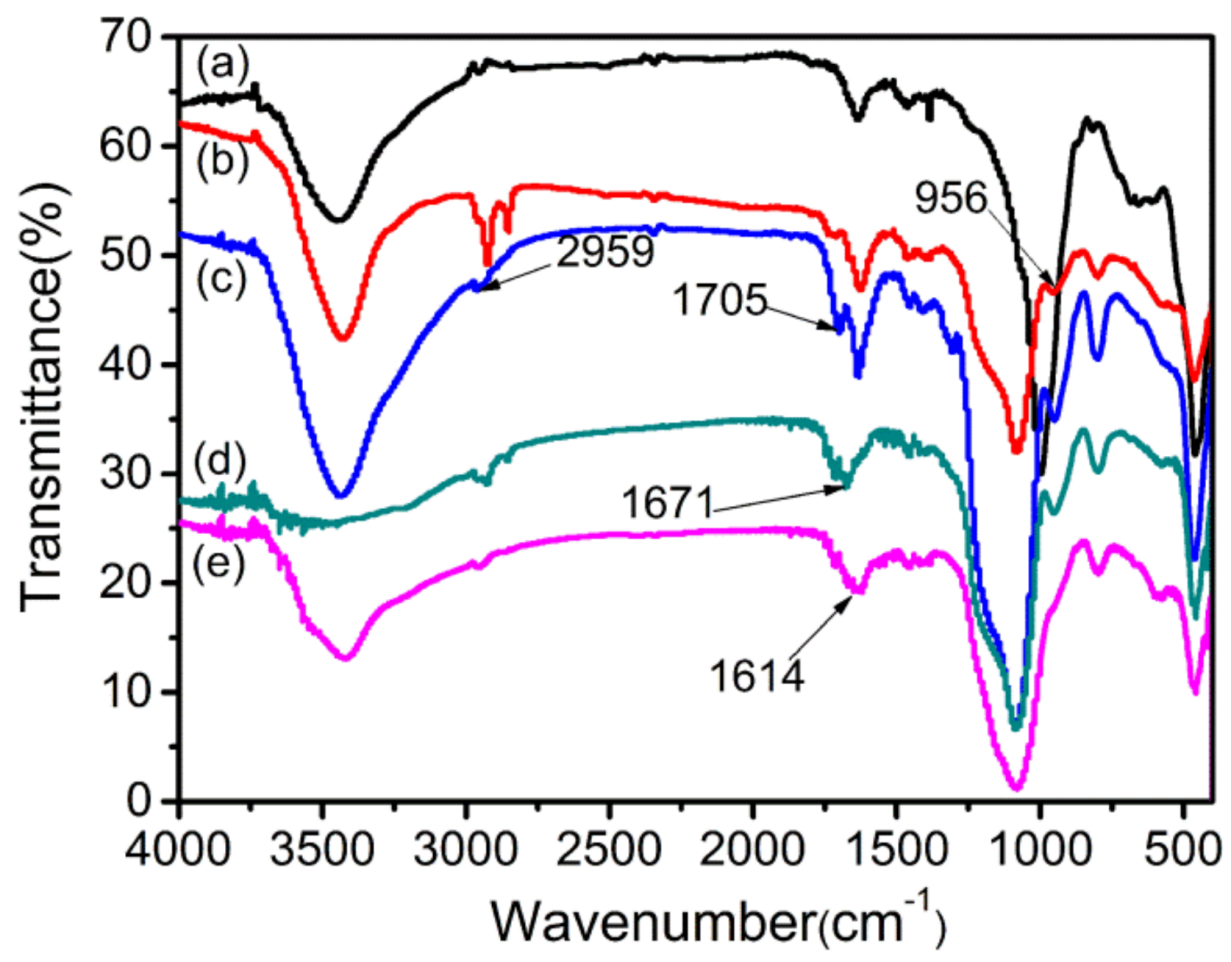
Fourier transform infrared (FTIR) spectra of (a) raw vermiculite (Verm), (b) acid-activated vermiculite (a-Verm), (c) 3-(triethoxysilyl)propyl methacrylate-modified acid vermiculites (OVerm), (d) polyacrylamide (PAM)/OVerm and (e) triethylenetetramine-grafted polyacrylamide (g-PAM)/OVerm.

Figure 4 shows the FTIR spectra of raw Verm, a-Verm, OVerm, PAM/OVerm and g-PAM/OVerm. Compared with the raw Verm, the Si–O stretching band gradually shifted from the characteristic position at 1010 cm^−1^, for tetrahedral, to 1081 cm^−1^. The peak at 1081 cm^−1^ is associated with Si–O stretching vibrations for amorphous silica with a three-dimensional framework, which were created on the surface of a-Verm by the simple acid treatment [45]. Moreover, the silanolization of the acid-treated product was verified by a new peak at 956 cm^−1^ caused by the stretching vibration of Si–OH groups exposed on the surface of a-Verm. The spectrum of OVerm shows a new band at 956 cm^−1^ for a-Verm (Figure 4b), which is usually representative of the silicon hydroxyl group (Si–OH). This suggests that new silanol groups (Si–OH) appeared on the surface of raw Verm after HCl modification. Absorption bands at 2959 cm^−1^ and 1705 cm^−1^ were ascribed to –C–H stretching vibrations and –COO– stretching vibrations of TEPM [46], respectively. These peaks suggest the successful modification of a-Verm with the silane coupling agent. The new absorption bands at 1671 cm^−1^ and 1614 cm^−1^ were assigned to C=O stretching vibrations and –NH_2_ bending vibrations, respectively, in the spectrum of the PAM/OVerm sample. These peaks confirmed the existence of PAM chains. Additionally, the absence of the absorption band at 1705 cm^−1^ was possibly due to surface coverage of PAM on OVerm. After the Mannich reaction, the peak around 1614 cm^−1^ in the spectrum of g-PAM/OVerm increased, indicating that triethylenetetramine was successfully grafted onto PAM through copolymerization.

#### 3.2.2. SEM and EDS Analyses

**Figure 5:**
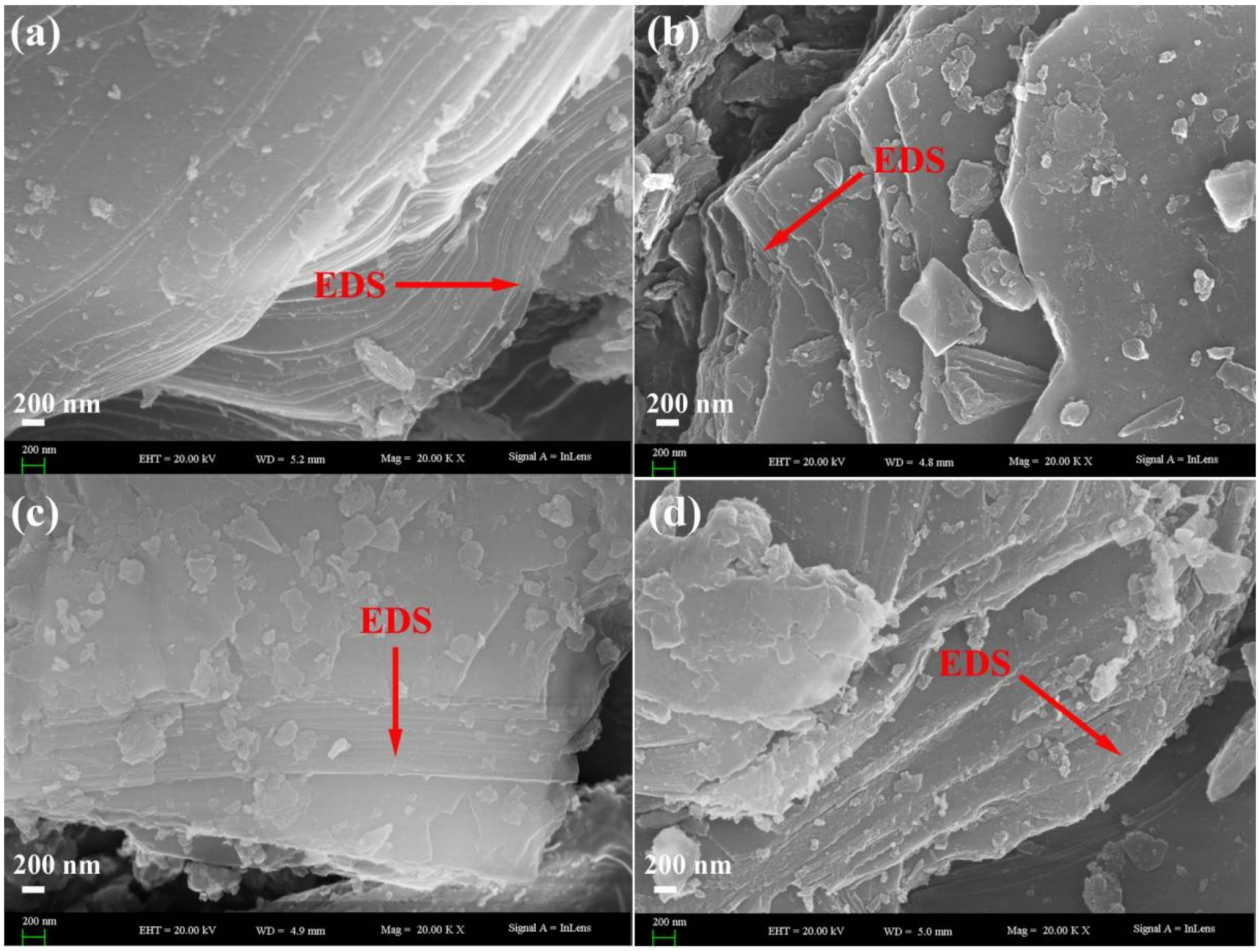
Scanning electron microscopy (SEM) images of (**a**) a-Verm; (**b**) OVerm; (**c**) PAM/OVerm; (**d**) g-PAM/OVerm.

The SEM images given in Figure 5 show a general view on the surface morphology of a-Verm, OVerm, PAM/OVerm and g-PAM/OVerm. From Figure 5a, Verm has a typical lamellar structure and a morphology containing fragmental shape and small particles, which is different from the g-PAM/OVerm morphology. The surface of Verm appears looser and more porous in comparison with OVerm, PAM/OVerm and g-PAM/OVerm, and no obvious protuberances were observed. With further modification and –NH_2_ grafting, the protuberances become more and more obvious (Figure 5b–d), which demonstrates the successful polymerization of AM and –NH_2_ grafting. Despite the appearance of more and more protuberances, the lamellar structure of the Verm samples remained unchanged, appearing relatively uniform and smooth. This supports the fact that chemical modification occurred on the surface of Verm, without destroying the basic crystal structure of Verm [45]. The SEM observations further confirmed the PAM modification and –NH_2_ grafting reaction on Verm.

**Figure 6:**
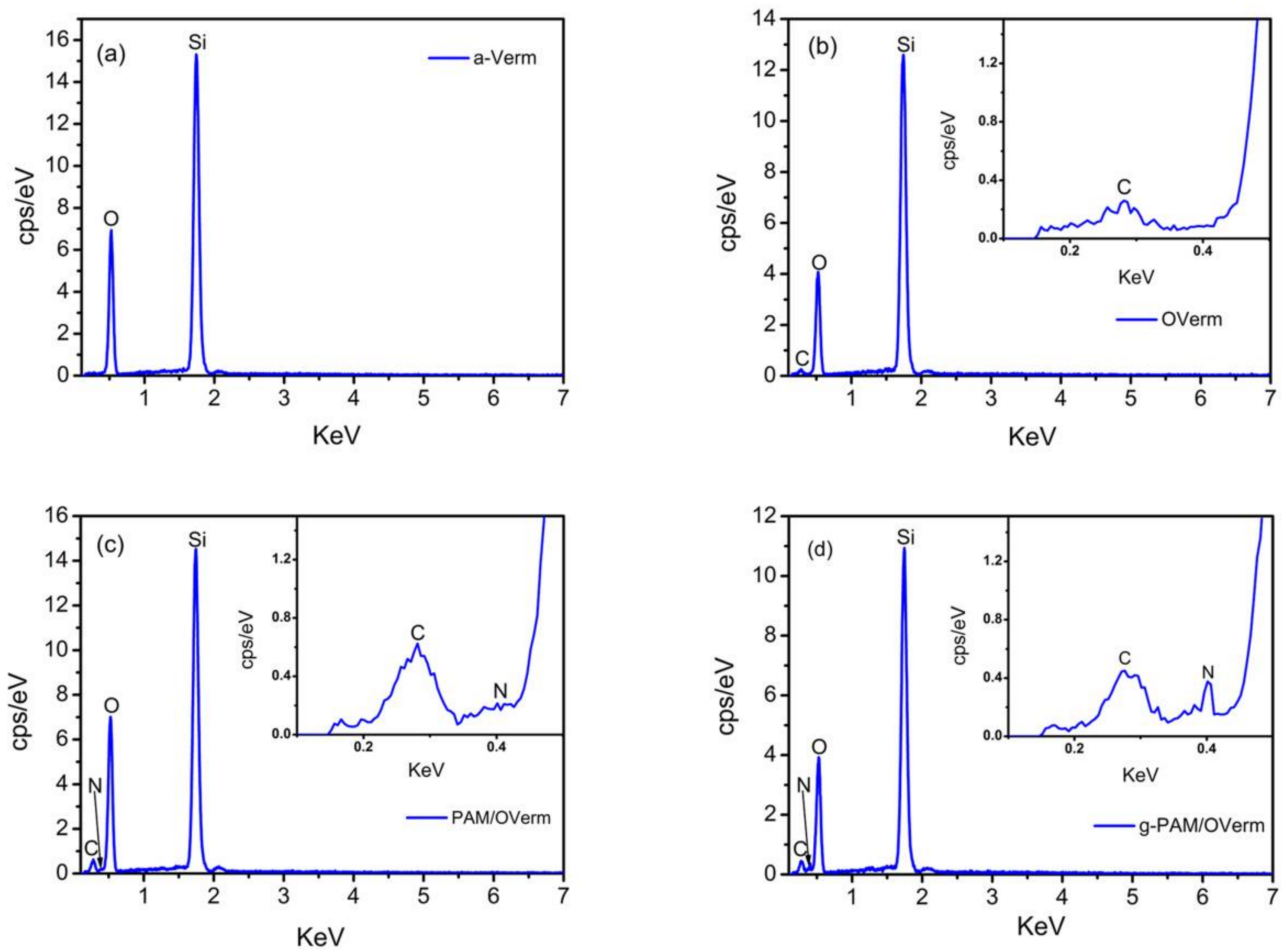
Energy dispersive spectrometer (EDS) patterns of (**a**) a-Verm; (**b**) OVerm; (**c**) PAM/OVerm; (**d**) g-PAM/OVerm.

**Figure 7:**
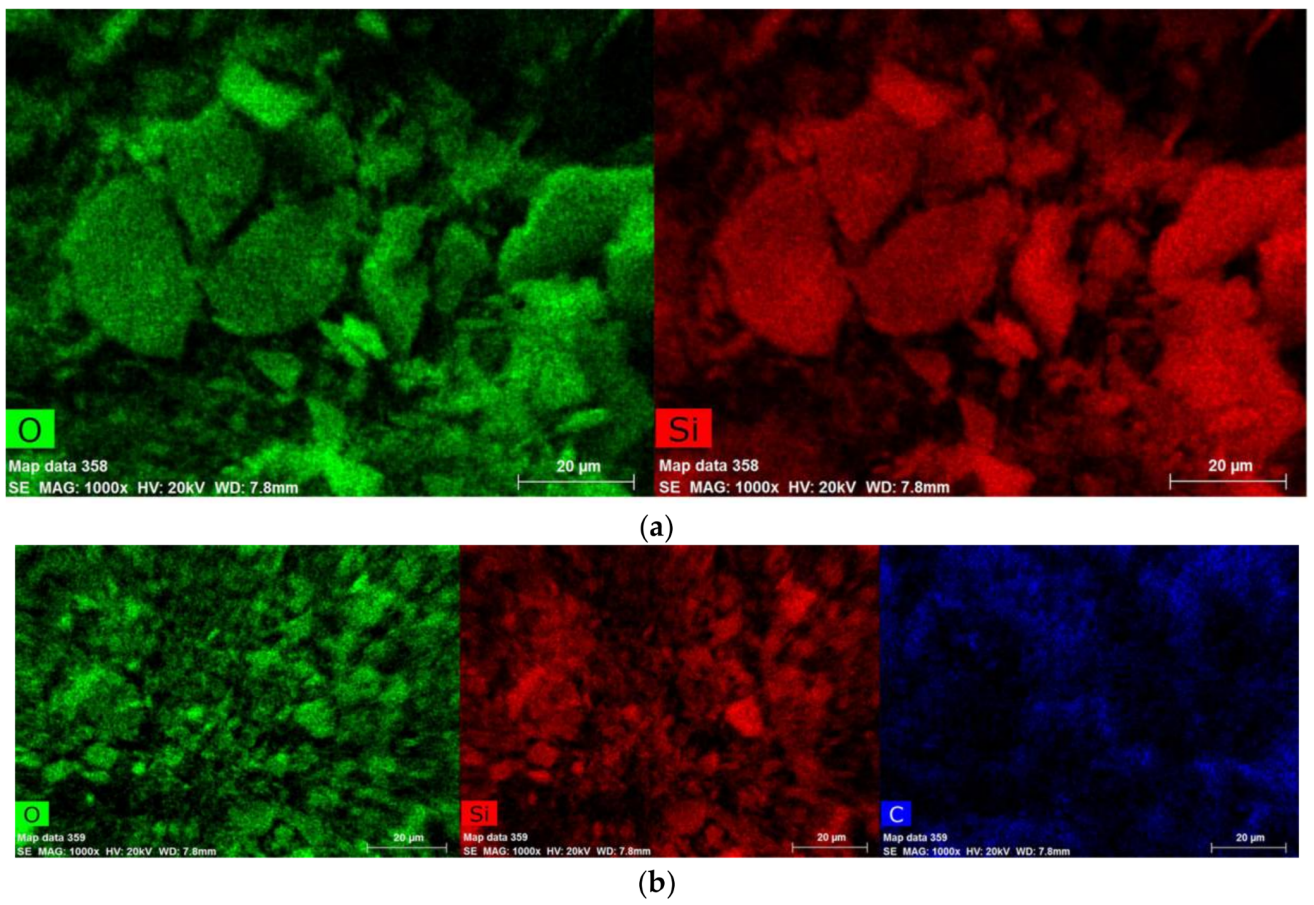

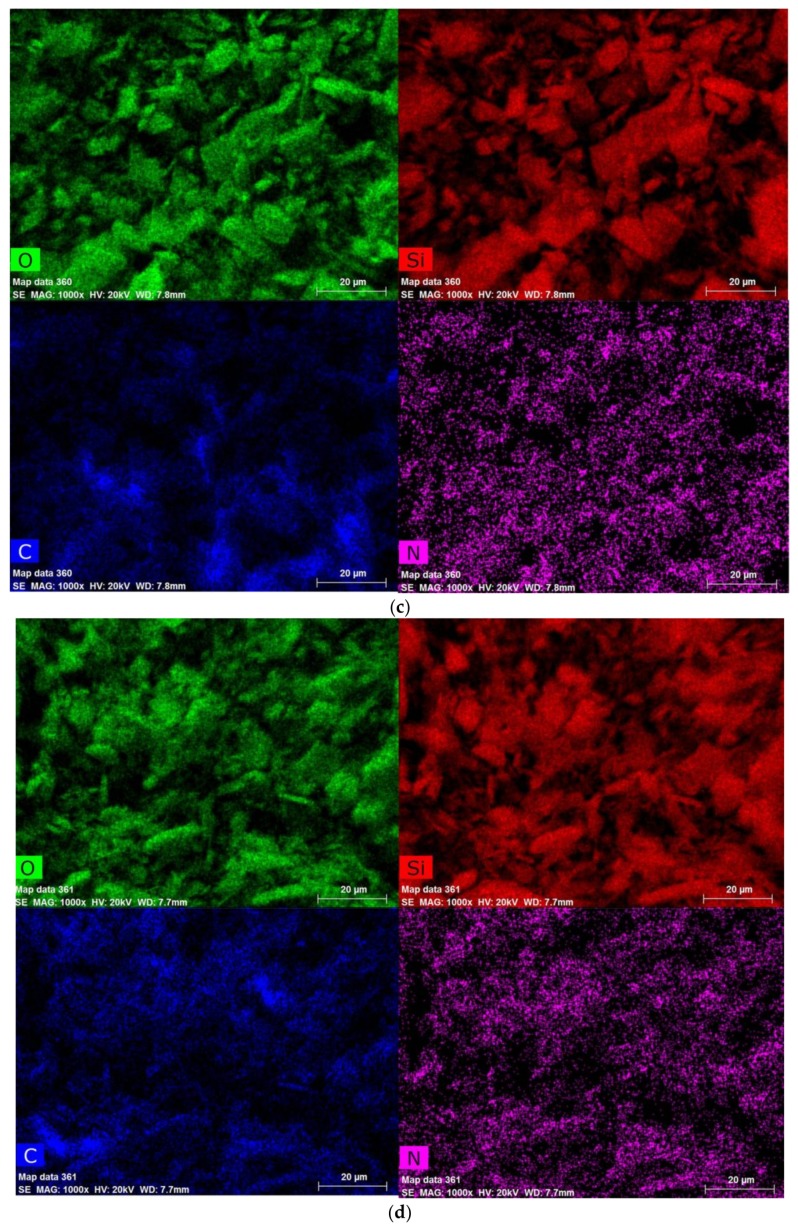
Elements analysis by mapping in SEM: (**a**) a-Verm; (**b**) OVerm; (**c**) PAM/OVerm; (**d**) g-PAM/OVerm.

EDS patterns of a-Verm, OVerm, PAM/OVerm and g-PAM/OVerm are displayed in Figure 6, which were obtained from the zones indicated in the SEM figures. The EDS spectra of a-Verm showed a dominant presence of Si and O, which originated from the frame of SiO_2_. After the organic modification, a small C peak was detected in OVerm, indicating that TEPM was successfully introduced into OVerm. The presence of elemental N and the increased C peak in PAM/OVerm give strong evidences for the successful immobilization of PAM on an organically modified Verm surface immediately after polymer formation (in situ immobilization). The peak of elemental N in g-PAM/OVerm doubled in height, in comparison with that of PAM/OVerm (Figure 6d). This confirmed the successful grafting of –NH_2_ onto the PAM chain. The semi-quantitative EDS results are listed in Table 1, and these results are in agreement with the FTIR measurements. Furthermore, Figure 7 shows the fundamental chemical elements existed in a-Verm, OVerm, PAM/OVerm and g-PAM/OVerm, such as Si, O, C and N element, which indicates the existence of chemical elements and the homogeneous element-distribution in the corresponding samples.

#### 3.2.3. Thermal Analysis

**Figure 8:**
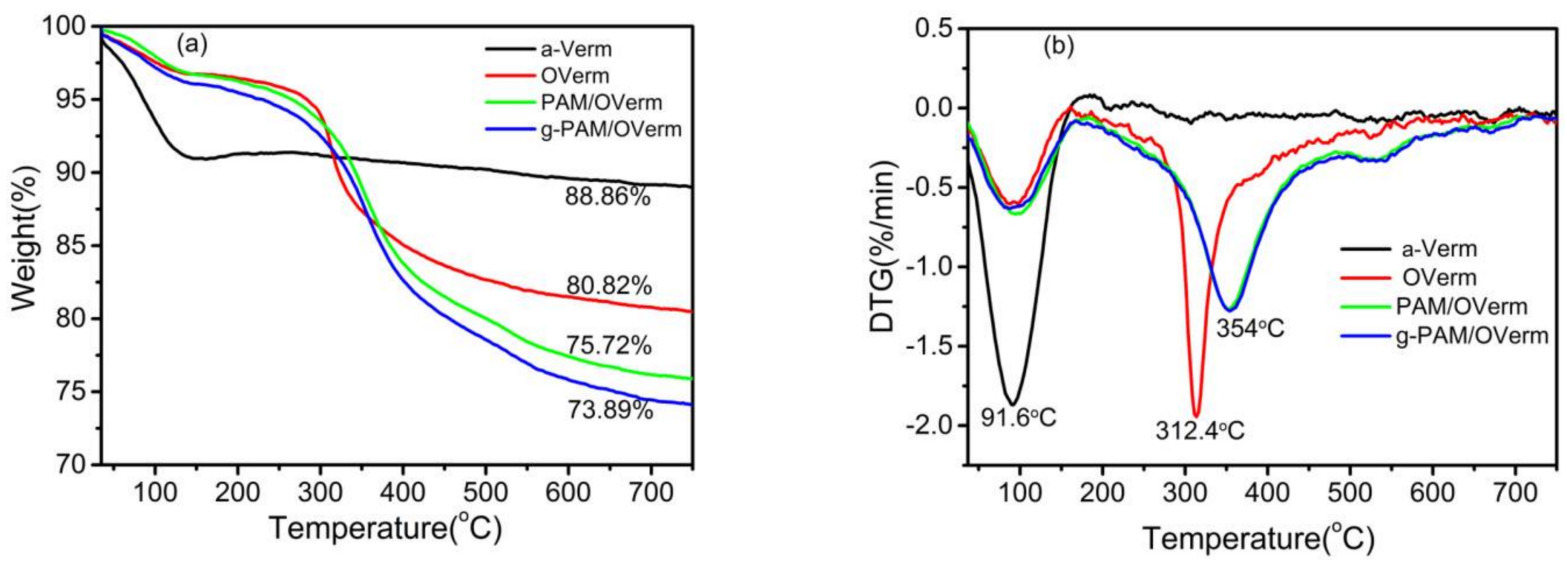
(**a**) TG and (**b**) DTG curves of a-Verm, OVerm, PAM/OVerm and g-PAM/OVerm.

The DTG curves of Verm samples (Figure 8b) show an endothermic peak at about 91.6 °C, implying a loss of moisture. The OVerm (TEPM-modified Verm) shows an endothermic peak at 312.4 °C, which was attributed to the decomposition of TEPM [31]. Moreover, the DTG curves of PAM/OVerm and g-PAM/OVerm also show an endothermic peak at 354 °C, largely caused by the decomposition of polyacrylamide . Moreover, TG curves (Figure 8a) show that the total mass loss of a-Verm, OVerm, PAM/OVerm and g-PAM/OVerm at 25–750 °C were 11.14%, 19.18%, 24.28% and 26.11%, respectively. The mass loss demonstrates that the number of organic groups including –C–H and –NH_2_ remaining in the samples per unit mass increased as the modification and grafting reactions proceeded. In other words, the TG and DTG analysis results confirmed the presence of the PAM modification and –NH_2_ grafting reaction on vermiculite.

#### 3.2.4. Nitrogen Adsorption–Desorption Isotherms

**Figure 9:**
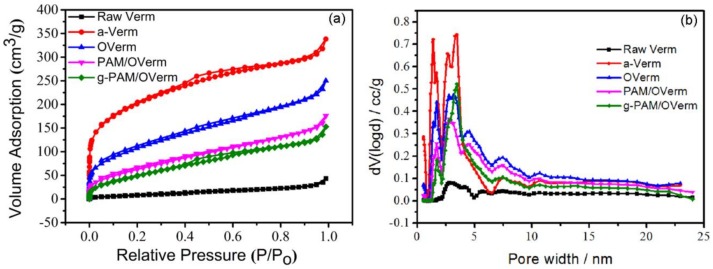
(**a**) N_2_ adsorption–desorption isotherms and (**b**) pore width distribution of Raw Verm, a-Verm, OVerm, PAM/OVerm and g-PAM/OVerm.

Nitrogen adsorption–desorption isotherms for vermiculite samples (Figure 9a) exhibit type II isotherms with a hysteresis loop, based on the IUPAC classification [47]. This indicates the existence of a large proportion of mesopores, with pore size between 2 nm and 50 nm. The hysteresis loop was type H3, due to the slit-shaped pores or plate-like particles [48]. The isotherm type for all vermiculite samples was the same. Figure 9b shows the pore width distribution of the vermiculite samples, suggesting that the pore width of vermiculite samples are distributed in the range of mesopore and micropore with the absence of macropore.

Table 2 shows surface area (S_BET_), pore volume and average pore diameter (Dp,a) of Raw Verm, a-Verm, OVerm, PAM/OVerm and g-PAM/OVerm. The surface area (S_BET_) increased after acid treatment. Raw Verm and a-Verm had an S_BET_ of 27.7 and 694.2 m^2^/g, respectively. The surface areas of OVerm, PAM/OVerm and g-PAM/OVerm were 396.4 m^2^/g, 247.3 m^2^/g and 201.8 m^2^/g, respectively. S_BET_ decreased with increasing modification and grafting reactions. The smaller S_BET_ of functionalized Verm was a consequence of the surface coverage of the silane coupling agent and polyacrylamide, which reduced the adsorption of N_2_. The same conclusion can be obtained by the changes of pore volume.

### 3.3. Adsorption of Pb(II)

#### 3.3.1. The Effect of pH on Pb(II) Uptake

**Figure 10:**
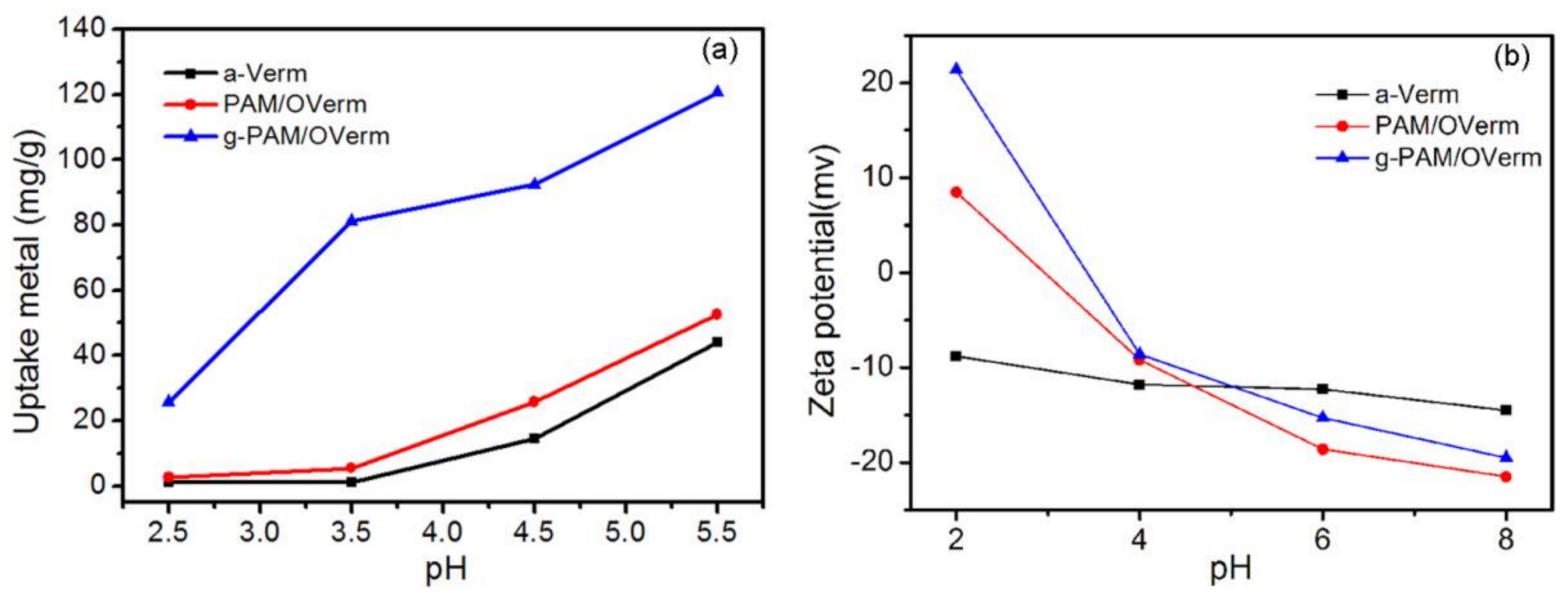
(**a**) Effect of pH on adsorption of Pb(II) (Reaction conditions: *C*_i_ = 400 mg·L^−1^, contact time t = 120 min, 0.05 g adsorbent); and (**b**) effect of solution pH on the zeta potential of a-Verm, PAM/OVerm and g-PAM/OVerm.

One of the main parameters affecting the adsorption process is the pH of the aqueous solution [49]. The effects of initial pH on the adsorption capacity of Pb(II) by a-Verm, PAM/OVerm and g-PAM/OVerm were studied individually, within the pH range of 2.5–5.5 (Figure 10a). Throughout the studied pH range, precipitation of Pb(II) did not occur. The adsorption capacity of Pb(II) ions increased with increasing initial pH, and maximum adsorption was achieved for an initial pH value of 5.5. Compared with a-Verm and PAM/OVerm, g-PAM/OVerm exhibited much higher adsorption capacity, which indicates that more –NH_2_ groups were successfully grafted onto PAM/OVerm. In addition, Pb(II) adsorption on g-PAM/OVerm was mainly due to the chelation between Pb(II) and the –NH_2_ groups. Furthermore, the amine groups were deprotonated, and the Pb(II)-amide linkage formation increased with increasing initial pH [36].

The Zeta potential of a-Verm, PAM/OVerm and g-PAM/OVerm as function of solution pH is shown in Figure 10b. With the increasing of pH vaule, the Zeta potential decreases. The Zeta potential values for the surface of a-Verm over the entire pH range from 2–8, which can be ascribed to the hydroxyl groups located at the surfaces of a-Verm [50]. Especially, positively charged surface of PAM/OVerm and g-PAM/OVerm can be observed when the pH value was lower than 4, which can be attributed the protonation of the –NH_2_ groups [51]. Compared with PAM/OVerm, the Zeta potential value of g-PAM/OVerm is more positive, suggesting that more –NH_2_ groups were grafted onto PAM/OVerm by the Mannich reaction. In addition, the Pb(II) adsorption sites might be occupied by a large amount of hydrogen ions when the pH value is ~2, making the adsorption quantity relatively low. In addition, g-PAM/OVerm shows stronger adsorption ability for Pb(II) compared with a-Verm and PAM/OVerm, which might be due to the larger quantity of –NH_2_ functional groups in g-PAM/OVerm.

#### 3.3.2. Study on the Adsorption Selectivity by g-PAM/OVerm

**Figure 11:**
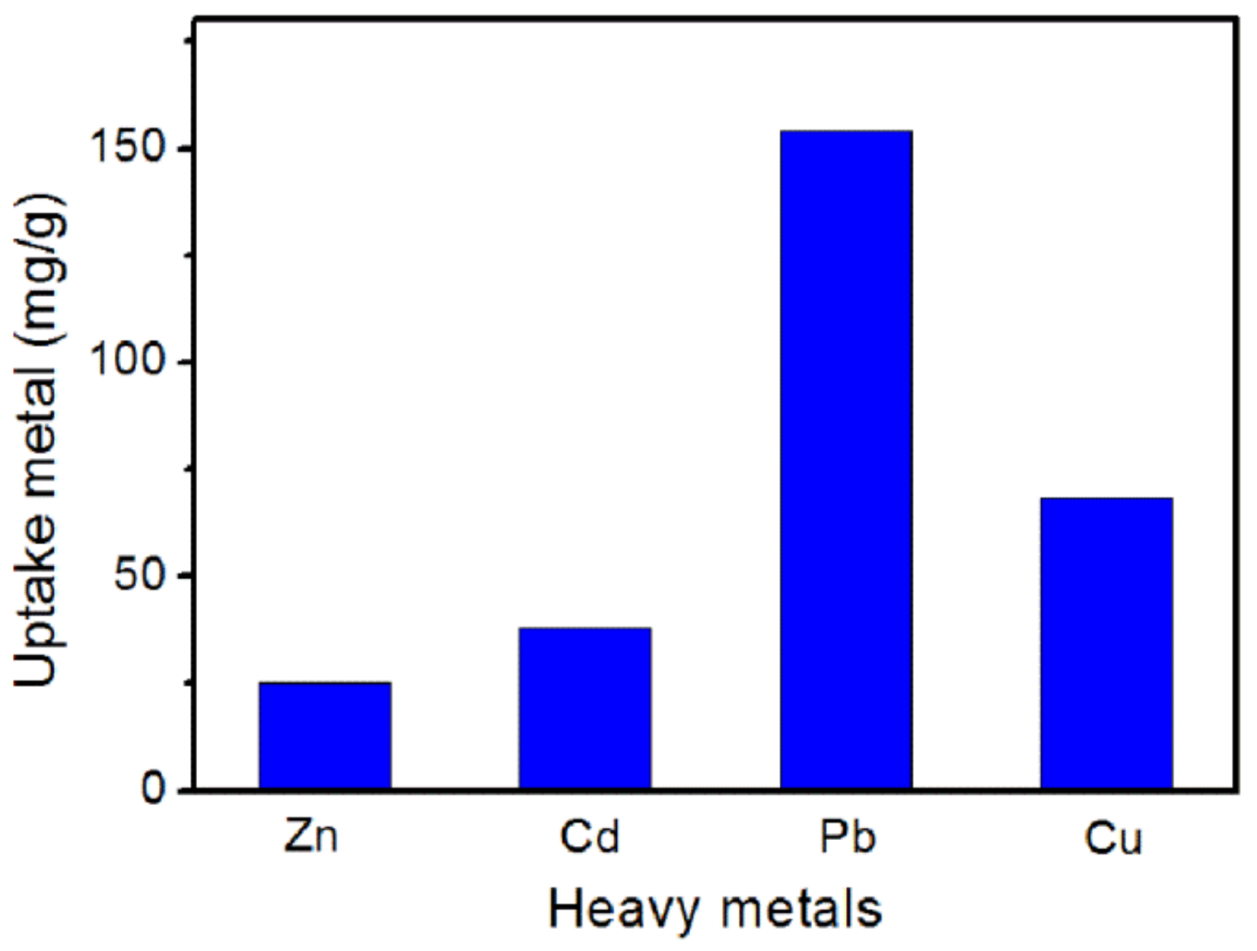
Effect of the presence of competitor ions on adsorption of Pb(II) (Reaction conditions: contact time t = 120 min, pH = 5.5, T = 30 °C).

To further verify whether g-PAM/OVerm have special adsorption selectivity for Pb(II) ions, a multi-component adsorption experiment was carried out. The initial concentrations of Zn(II), Cd(II), Pb(II) and Cu(II) are all 800 mg·L^−1^. Results of selective adsorption experiments of the nanocomposites for heavy metal ions in mixed solutions are shown in Figure 11. The adsorption capacities of g-PAM/OVerm for Zn(II), Cd(II), Pb(II) and Cu(II) ions are 25.3, 37.8, 153.8 and 68.3 mg·g^−1^, respectively. The adsorption capacity for Pb(II) ion is much higher than that for Zn(II), Cd(II) and Cu(II) ions, suggesting that g-PAM/OVerm has better selectivity for Pb(II) ion in comparison with Zn(II), Cd(II) and Cu(II) ions under the same condition, which can be attributed to the stronger affinity between Pb(II) ion and –NH_2_ groups of g-PAM/OVerm [52,53].

#### 3.3.3. g-PAM/OVerm Regeneration

**Figure 12:**
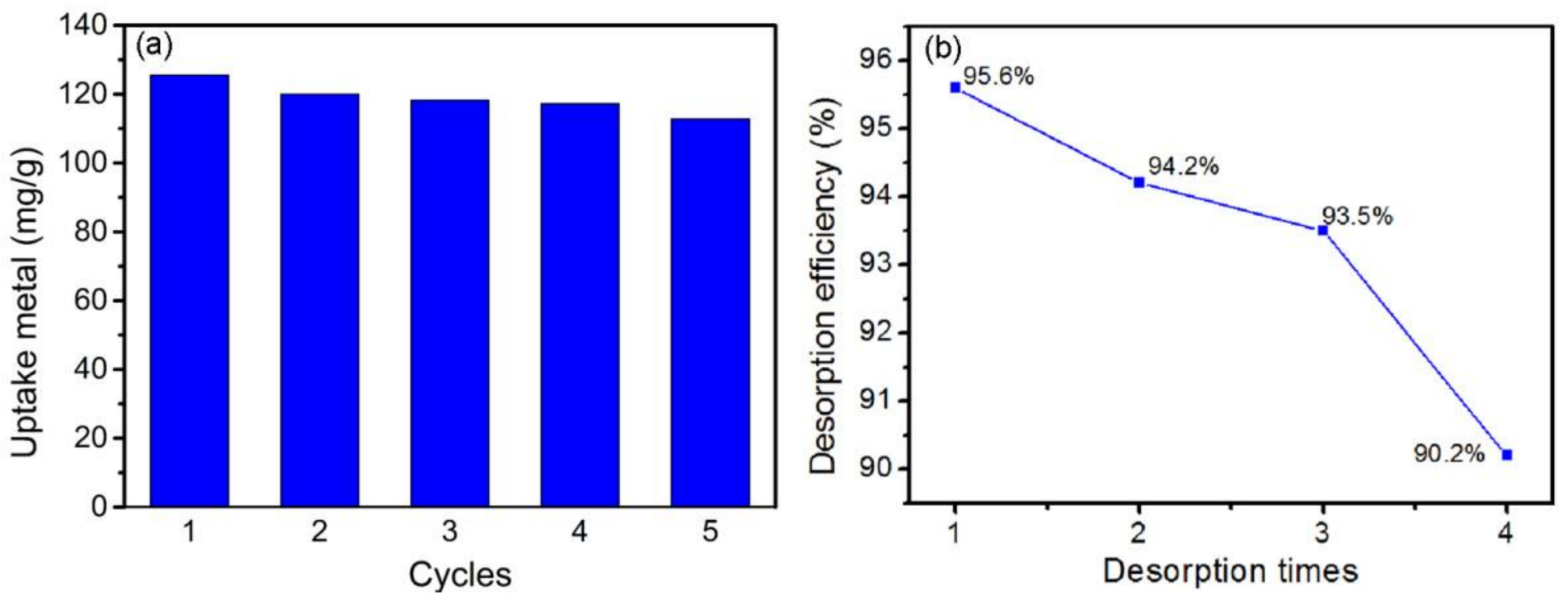
(**a**) Regeneration studies of g-PAM/OVerm after five cycles; and (**b**) evolution of the desorption efficiencies during four-time desorption.

The regeneration of g-PAM/OVerm for Pb(II) adsorption was performed in a batch experiment. Hydrochloric acid was used to desorb the Pb(II) from the adsorbents. Samples were kept in hydrochloric acid for 30 min in a constant temperature vibration shaker. After centrifugation, the supernatant was discarded and the g-PAM/OVerm was reused. Figure 12a reveals the Pb(II) adsorption capacity by g-PAM/OVerm during five cycles of adsorption/desorption, and Figure 12b shows the evolution of the desorption efficiencies during four-time desorption calculated from the five cycles of adsorption/desorption. The regenerated g-PAM/OVerm maintained 90% of its adsorption capacity after the fifth test cycle and 90.2% of its desorption efficiency after the fourth desorption process, which suggests that g-PAM/OVerm adsorbents are promising for the efficient adsorption of Pb(II).

#### 3.3.4. Adsorption Dynamics

**Figure 13:**
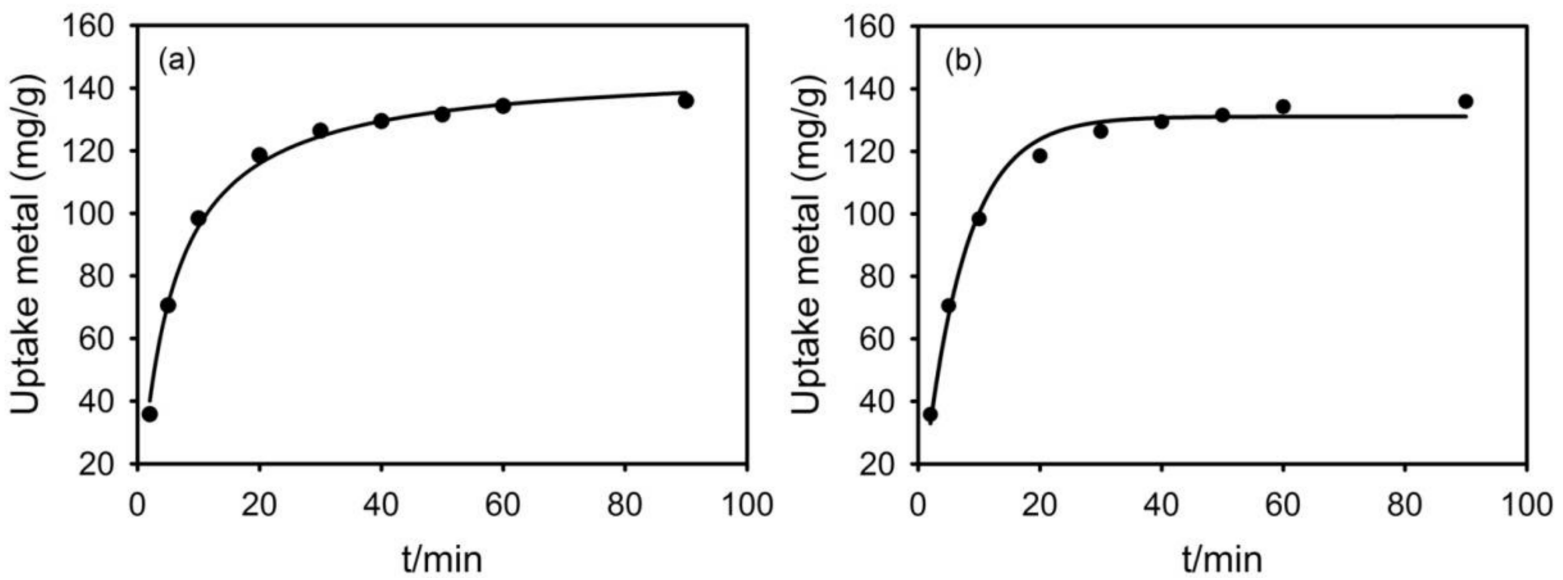
(**a**) The pseudo-second-order kinetic model, (**b**) the pseudo-first-order kinetic model for the adsorption of Pb(II) by g-PAM/OVerm (Reaction conditions: initial suspension pH = 5.5, 0.05 g g-PAM/OVerm, T = 30 °C).

The effects of time on the adsorption of Pb(II) by g-PAM/OVerm are given in Figure 13a,b. The adsorption rate was very fast during the first 20 min, and the removal of Pb(II) was 120.1 mg·g^−1^. After that, the adsorption rate was very slow, and the adsorption equilibrium for Pb(II) was reached within 60 min.

For the purpose of determining the reaction mechanisms and mass transfer controlling Pb(II) removal by the g-PAM/OVerm adsorbent, pseudo-second-order and pseudo-first-order kinetic equations were used to analyze the experimental data. The pseudo-first-order kinetic model can be expressed in a nonlinear form as [54,55]:

(2) $$q_{t}=q_{e}\left(1{-e}^{{-k}_{1}t}\right),$$

where q_t_ and q_e_ are the Pb(II) uptake amounts (mg·g^−1^) by the adsorbent g-PAM/OVerm at time t and at equilibrium; k_1_ (min^−1^) is the rate constant of the pseudo-first-order adsorption. The values of k_1_, q_e_ and the correlation coefficient (r^2^) were determined from the nonlinear plot of q_t_ versus t (Figure 13a). The pseudo-second-order kinetic model can be expressed from the following equation [56,57]:

(3) $$\frac{t}{q_{t}}=\frac{1}{k_{2}q_{e}^{2}}+\frac{t}{q_{e}}.$$

Equation (3) can be converted into the nonlinear form (Equation (4)), which has the ability to predict the adsorption equilibrium and form an intuitive contrast with the pseudo-first-order kinetic model:

(4) $$q_{t}=\frac{q_{e}^{2}k_{2}t}{1+q_{e}k_{2}t}.$$

where k_2_ (g·mg^−1^·min^−1^) is the rate constant of pseudo-second-order kinetic model. The constants can be calculated from plotting (t/q_t_) versus t. Figure 13b presents the plot of q_t_ versus t for the adsorption of Pb(II) onto g-PAM/OVerm in the form of a nonlinear plot. Table 3 presents the values of k, q_e_ and the correlation coefficient (r^2^) of the pseudo-first-order and pseudo-second-order kinetic models.

From Table 3, the correlation coefficient of the pseudo-first-order model was relatively low, showing a poor fit to the adsorption data. However, the correlation coefficient for the pseudo-second-order was more than 0.995. The calculated q_e_ value was very close to the experimental data, according to pseudo-second-order kinetic model. The adsorption dynamics analysis suggests that the Pb(II) adsorption kinetics fit the pseudo-second-order model. Therefore, the pseudo-second-order mechanism was determined as the predominant kinetic process of Pb(II) adsorption by g-PAM/OVerm, which corresponds to chemical adsorption as the rate-controlling step [57].

#### 3.3.5. Adsorption Isotherms

The effects of initial concentration and adsorption capacity on Pb(II) removal were investigated by adding 50 mg of g-PAM/OVerm into 25 mL of Pb(II) solutions, with a range of Pb(II) concentrations, for 6 h at 30 °C. Langmuir and Freundlich isotherm models (Equations (5) and (6)) were carried out to explore the Pb(II) adsorption equilibrium [58,59]:

(5) $$q_{e}=\frac{q_{m}K_{L}C_{e}}{1+K_{L}C_{e}},$$

(6) $$q_{e}=K_{F}C_{e}^{\frac{1}{n}}.$$

Here, q_m_ is the maximum capacity of adsorption (mg·g^−1^), q_e_ is the equilibrium adsorption capacity (mg·g^−1^) for Pb(II), *C*_e_ is the Pb(II) concentration at equilibrium in the solution (mg·L^−1^), *K_L_* and *K_F_* are the Langmuir and Freundlich isotherm equilibrium constants (L·mg^−1^), respectively.

**Figure 14:**
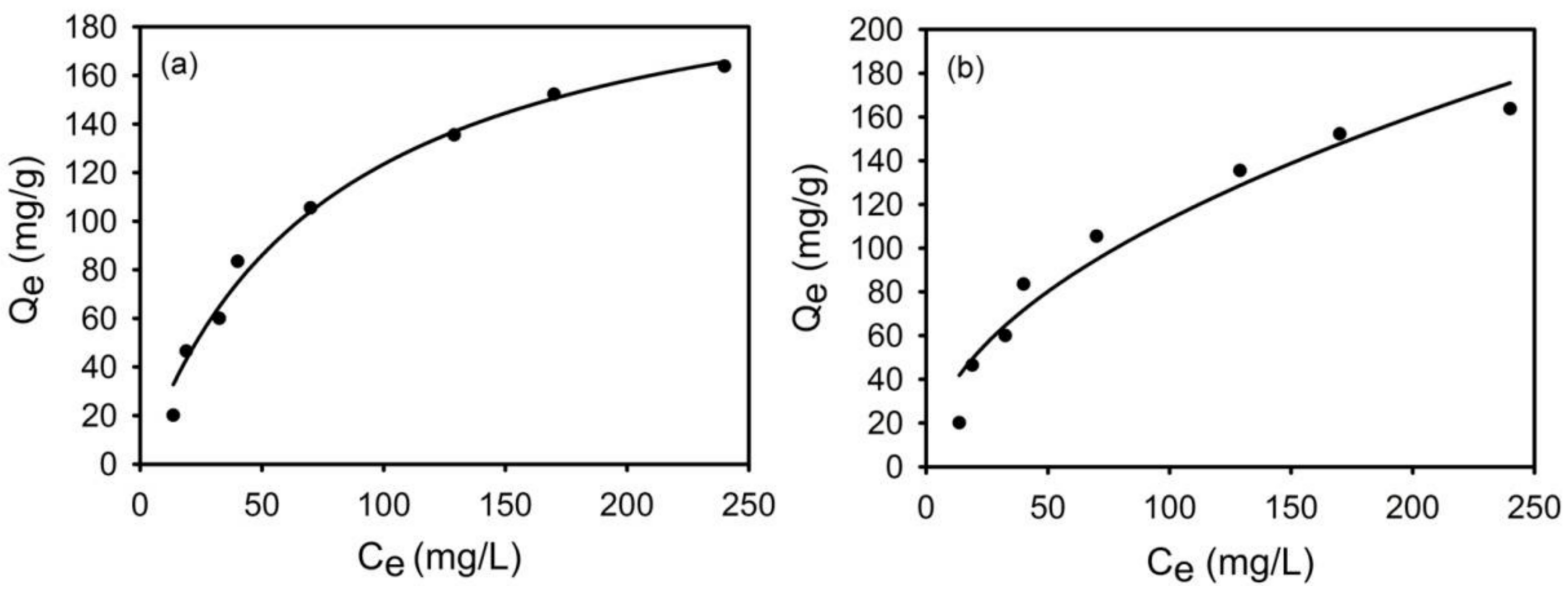
(**a**) Langmuir plot and (**b**) Freundlich plot for the adsorption of Pb(II) by g-PAM/OVerm (Reaction conditions: initial suspension pH = 5.5, 0.05 g g-PAM/OVerm, T = 30 °C).

The results are shown in Figure 14a,b and Table 4. In general, Pb(II) adsorption by g-PAM/OVerm increased with increasing initial Pb(II) concentrations, and the correlation coefficient values suggest the Langmuir model better represents the adsorption isotherm, than the Freundlich model. This indicates that the monolayer adsorption mechanism describes the adsorption of Pb(II) because the active sites are distributed evenly on the surface of g-PAM/OVerm [60]. Based on the Langmuir equation, the theoretical maximum adsorption capacity (q_m_) of Pb(II) on g-PAM/OVerm was 219.4 mg·g^−1^. The results obtained from this study were found to be higher than that of many corresponding adsorbents reported in the literature (Table 5), revealing that modified vermiculite is suitable for the removal of Pb(II) ions from aqueous solutions. Therefore, considering the economic advantage and the adsorption capacity, g-PAM/OVerm is a very potential adsorbent for Pb(II) ions.

### 3.4. Pb(II) Adsorption Mechanism

**Figure 15:**
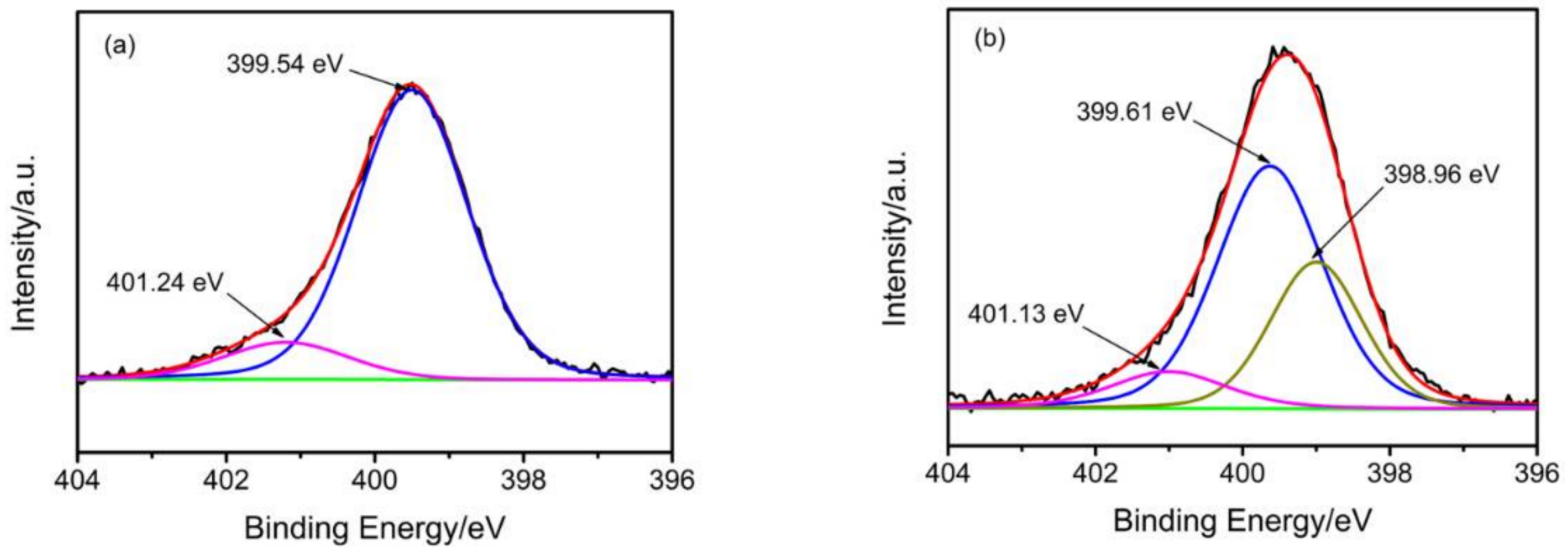
XPS N 1s spectra of g-PAM/OVerm: (**a**) before Pb(II) adsorption and (**b**) after Pb(II) adsorption.

For the purpose of determining the adsorption mechanism, high resolution XPS spectra analyses were carried out for g-PAM/OVerm before and after adsorption of Pb(II). Figure 15 reveals the characteristic N 1s spectra of g-PAM/OVerm before and after Pb(II) adsorption. Before Pb(II) adsorption, two peaks, including one weak and one strong, were found in the N 1s spectra at the binding energy (BE) of about 401.24 and 399.54 eV, respectively (Figure 15a). The strong peak was attributed to nitrogen atoms of the –NH_2_ groups in the grafted TETA. The weaker peak at 401.24 eV was attributed to nitrogen atoms of the O=C–NH_2_ groups in PAM, indicating that the number of –NH_2_ groups in the grafted TETA was far greater than that in PAM [33]. However, one more peak appeared at 398.96 eV in the N 1s spectra after Pb(II) adsorption (Figure 15b). The intensity of the new peak is very strong relatively, indicating that a number of nitrogen atoms existed in a more reduced state after Pb(II) adsorption [32]. This resulted from the formation of the covalent bond between –NH_2_ and Pb. Thus, the density of the electron cloud for the nitrogen atom increased, decreasing the BE of the peak. Therefore, the XPS analyses supported the formation of covalent bonds between amide groups and Pb(II) as the adsorption mechanism.

## 4. Conclusions

In this work, an effective approach to functionalization of Verm for removal of Pb(II) was developed by significantly improving specific surface area and silanol groups of Verm by acid activation and further modifying Verm surface with a series of organic reactions for introducing more functional amine groups. By silane coupling agent modification, polymerization of AM and –NH_2_-grafting Mannich reaction, more nitrogen Lewis basic centers were created, thus significantly enhancing the Verm adsorption capacity for Pb(II). The amine-functionalized Verm showed much greater efficiency in Pb(II) adsorption at different pH values than unmodified Verm. Furthermore, g-PAM/OVerm has better selectivity for Pb(II) ion in comparison with Zn(II), Cd(II) and Cu(II) ions. The adsorption equilibrium data on g-PAM/OVerm were in good agreement with the Langmuir adsorption isotherms, and the adsorption maximal value of Pb(II) was 219.4 mg·g^−1^. The kinetics data fit the pseudo-second-order kinetic well. The great adsorption capacity of g-PAM/OVerm might be because of the presence of strong covalent bonds between Pb(II) and the –NH_2_ groups. This methodology can be expanded to activate the surfaces of various materials with different functional groups such as –COOH, –OH and –SH. It is concluded that the new amine-functionalized Verm could be used as a low cost and new effective adsorbent for removing Pb(II) from aqueous systems. The present work contributes to the development of novel cost-efficient adsorbents with great adsorption capacities and unique selectivity for different heavy metals.

## Figures and Tables

**Table 1:** Elemental Analysis of a-Verm, OVerm, PAM/OVerm and g-PAM/OVerm by EDS.

Samples	Elements	a-Verm	OVerm	PAM/OVerm	g-PAM/OVerm
Elements content (weight %)	O	58.3	50.9	55.8	50.8
	Si	41.7	38.2	27.1	28.5
	C	0	10.9	13.1	14.8
	N	0	0	4.0	5.9

**Table 2:** Specific surface area (S_BET_), pore volume and average pore diameter analysis of raw Verm, a-Verm, OVerm, PAM/OVerm and g-PAM/OVerm.

Samples	Raw Verm	a-Verm	OVerm	PAM/OVerm	g-PAM/OVerm
S_BET_ (m^2^/g)	27.7	694.2	396.4	247.3	201.8
V_total_ (m^3^/g)	6.7 × 10^−2^	5.2 × 10^−1^	3.8 × 10^−1^	2.7 × 10^−1^	2.4 × 10^−1^
V_meso_ (m^3^/g)	6.7 × 10^−2^	3.9 × 10^−1^	3.6 × 10^−1^	2.7 × 10^−1^	2.4 × 10^−1^
V_micro_ (m^3^/g)	0	1.3 × 10^−1^	1.9 × 10^−2^	0	0
D_p,a_ (nm)	10.9	3.0	3.9	4.4	4.7

**Table 3:** Parameters of kinetic models for Pb(II) adsorption onto g-PAM/OVerm.

Metal Ion	Pseudo-First-Order			Pseudo-Second-Order		
	q_e_ (mg·g^−1^)	k_1_ (min^−1^)	r^2^	q_e_ (mg·g^−1^)	k_2_ (g·mg^−1^·min^−1^)	r^2^
Pb^2+^	131.1	0.1447	0.9903	146.7	0.0012	0.9954

**Table 4:** Langmuir and Freundlich parameters for Pb(II) adsorption on g-PAM/OVerm.

Metal Ion	Langmuir			Freundlich		
	q_m_ (mg·g^−1^)	*K_L_* (L·mg^−1^)	r^2^	*K_F_* (mg^1−n^·L^n^·g^−1^)	n	r^2^
Pb^2+^	219.4	2.8	0.9851	11.3	1.9	0.9498

**Table 5:** Comparison of Pb(II) adsorption capacity on g-PAM/OVerm and other clay-based adsorbents.

Adsorbent	pH	T (°C)	q_m_ (mg·g^−1^)	Ref.
Triethylene tetramine (TETA)-montmorillonite	4.8	30	2.44	[61]
Chitosan-clay composite beads	4.5	25	7.93	[62]
Modified kaolinite clay	5.0	30	32.2	[63]
EMATP-modified attapulgite	5.0	30	158.0	[64]
Poly(acrylic acid)-bentonite	5.0	50	96.1	[65]
Mg2Al-DTPA LDH	5.5	30	170.0	[66]
Montmorillonite-TOA	7.0	25	33.1	[67]
Clay/poly(methoxyethyl)acrylamide	5.0	30	81.0	[68]
g-PAM/OVerm	5.5	30	219.4	This study

## References

[1] Fu F.L., Wang Q. (2011). Removal of heavy metal ions from wastewaters: A review. J. Environ. Manag..

[2] Deng J.Q., Liu Y.Q., Liu S.B., Zeng G.M., Tan X.F., Huang B.Y., Tang X.J., Wang S.F., Hua Q., Yan Z.L. (2017). Competitive adsorption of Pb(II), Cd(II) and Cu(II) onto chitosan-pyromellitic dianhydride modified biochar. J. Colloid Interface Sci..

[3] Uddin M.K. (2017). A review on the adsorption of heavy metals by clay minerals, with special focus on the past decade. Chem. Eng. J..

[4] Haider S., Park S.Y. (2009). Preparation of the electrospun chitosan nanofibers and their applications to the adsorption of Cu(II) and Pb(II) ions from an aqueous solution. J. Membr. Sci..

[5] Ju X.J., Zhang S.B., Zhou M.Y., Xie R., Yang L., Chu L.Y. (2009). Novel heavy-metal adsorption material: Ion-recognition P(NIPAM-co-BCAm) hydrogels for removal of lead(II) ions. J. Hazard. Mater..

[6] Jana S., Saikia A., Purkait M.K., Mohanty K. (2011). Chitosan based ceramic ultrafiltration membrane: Preparation, characterization and application to remove Hg(II) and As(III) using polymer enhanced ultrafiltration. Chem. Eng. J..

[7] Samiey B., Cheng C.H., Wu J.N. (2014). Organic-Inorganic Hybrid Polymers as Adsorbents for Removal of Heavy Metal Ions from Solutions: A Review. Materials.

[8] Zhang M.L., Zhang Z.H., Liu Y.N., Yang X., Luo L.J., Chen J.T., Yao S.Z. (2011). Preparation of core-shell magnetic ion-imprinted polymer for selective extraction of Pb(II) from environmental samples. Chem. Eng. J..

[9] Berber-Mendoza M.S., Leyva-Ramos R., Alonso-Davila P., Fuentes-Rubio L., Guerrero-Coronado R.M. (2006). Comparison of isotherms for the ion exchange of Pb(II) from aqueous solution onto homoionic clinoptilolite. J. Colloid Interface Sci..

[10] Veli S., Alyuz B. (2007). Adsorption of copper and zinc from aqueous solutions by using natural clay. J. Hazard. Mater..

[11] Goel J., Kadirvelu K., Rajagopal C., Garg V.K. (2005). Removal of lead(II) by adsorption using treated granular activated carbon: Batch and column studies. J. Hazard. Mater..

[12] Meena A.K., Mishra G.K., Rai P.K., Rajagopal C., Nagar P.N. (2005). Removal of heavy metal ions from aqueous solutions using carbon aerogel as an adsorbent. J. Hazard. Mater..

[13] Aguado J., Arsuaga J.M., Arencibia A., Lindo M., Gascon V. (2009). Aqueous heavy metals removal by adsorption on amine-functionalized mesoporous silica. J. Hazard. Mater..

[14] Chen A.H., Liu S.C., Chen C.Y., Chen C.Y. (2008). Comparative adsorption of Cu(II), Zn(II), and Pb(II) ions in aqueous solution on the crosslinked chitosan with epichlorohydrin. J. Hazard. Mater..

[15] Sen Gupta S., Bhattacharyya K.G. (2012). Adsorption of heavy metals on kaolinite and montmorillonite: A review. Phys. Chem. Chem. Phys..

[16] Sdiri A., Higashi T., Chaabouni R., Jamoussi F. (2012). Competitive Removal of Heavy Metals from Aqueous Solutions by Montmorillonitic and Calcareous Clays. Water Air Soil Pollut..

[17] Tjong S.C., Meng Y.Z., Hay A.S. (2002). Novel preparation and properties of polypropylene-vermiculite nanocomposites. Chem. Mater..

[18] Skipper N.T., Soper A.K., McConnell J.D.C. (1991). The structure of interlayer water in vermiculite. J. Chem. Phys..

[19] Temuujin J., Okada K., MacKenzie K.J.D. (2003). Preparation of porous silica from vermiculite by selective leaching. Appl. Clay Sci..

[20] Hashem F.S., Amin M.S., El-Gamal S.M.A. (2015). Chemical activation of vermiculite to produce highly efficient material for Pb^2+^ and Cd^2+^ removal. Appl. Clay Sci..

[21] Panuccio M.R., Sorgona A., Rizzo M., Cacco G. (2009). Cadmium adsorption on vermiculite, zeolite and pumice: Batch experimental studies. J. Environ. Manag..

[22] Stylianou M.A., Inglezakis V.J., Moustakas K.G., Malamis S.P., Loizidou M.D. (2007). Removal of Cu(II) in fixed bed and batch reactors using natural zeolite and exfoliated vermiculite as adsorbents. Desalination.

[23] Malandrino M., Abollino O., Giacomino A., Aceto M., Mentasti E. (2006). Adsorption of heavy metals on vermiculite: Influence of pH and organic ligands. J. Colloid Interface Sci..

[24] Jozefaciuk G., Bowanko G. (2002). Effect of acid and alkali treatments on surface areas and adsorption energies of selected minerals. Clays Clay Miner..

[25] Ravichandran J., Sivasankar B. (1997). Properties and catalytic activity of acid-modified montmorillonite and vermiculite. Clays Clay Miner..

[26] Yu X.B., Wei C.H., Ke L., Wu H.Z., Chai X.S., Hu Y. (2012). Preparation of trimethylchlorosilane-modified acid vermiculites for removing diethyl phthalate from water. J. Colloid Interface Sci..

[27] Fan Q.H., Shao D.D., Hu J., Wu W.S., Wang X.K. (2008). Comparison of Ni^2+^ sorption to bare and ACT-graft attapulgites: Effect of pH, temperature and foreign ions. Surf. Sci..

[28] Liu P., Wang T.M. (2007). Adsorption properties of hyperbranched aliphatic polyester. grafted attapulgite towards heavy metal ions. J. Hazard. Mater..

[29] Chang Y., Liu H.W., Zha F., Chen H.K., Ren X.N., Lei Z.Q. (2011). Adsorption of Pb(II) by *N*-methylimidazole modified palygorskite. Chem. Eng. J..

[30] Huang J.H., Liu Y.F., Wang X.G. (2008). Selective adsorption of tannin from flavonoids by organically modified attapulgite clay. J. Hazard. Mater..

[31] Chen Y., Zhao Y., Zhou S., Chu X., Yang L., Xing W. (2009). Preparation and characterization of polyacrylamide/palygorskite. Appl. Clay Sci..

[32] Xue A.L., Zhou S.Y., Zhao Y.J., Lu X.P., Han P.F. (2011). Effective NH_2_-grafting on attapulgite surfaces for adsorption of reactive dyes. J. Hazard. Mater..

[33] Zhao Y., Chen Y., Li M., Zhou S., Xue A., Xing W. (2009). Adsorption of Hg^2+^ from aqueous solution onto polyacrylamide/attapulgite. J. Hazard. Mater..

[34] Liu P. (2007). Polymer modified clay minerals: A review. Appl. Clay Sci..

[35] Manju G.N., Krishnan K.A., Vinod V.P., Anirudhan T.S. (2002). An investigation into the sorption of heavy metals from wastewaters by polyacrylamide-grafted iron(III) oxide. J. Hazard. Mater..

[36] Bicak N., Sherrington D.C., Senkal B.F. (1999). Graft copolymer of acrylamide onto cellulose as mercury selective sorbent. React. Funct. Polym..

[37] Liu P., Guo J.S. (2006). Polyacrylamide grafted attapulgite (PAM-ATP) via surface-initiated atom transfer radical polymerization (SI-ATRP) for removal of Hg(II) ion and dyes. Colloids Surf. A Physicochem. Eng. Asp..

[38] Sayin S., Ozcan F., Yilmaz M., Tor A., Memon S., Cengeloglu Y. (2010). Synthesis of Calix 4 arene-grafted Magnetite Nanoparticles and Evaluation of Their Arsenate as Well as Dichromate Removal Efficiency. Clean Soil Air Water.

[39] Kasgoz H., Ozgumus S., Orbay M. (2003). Modified polyacrylamide hydrogels and their application in removal of heavy metal ions. Polymer.

[40] Ge Y.Y., Li Z.L., Kong Y., Song Q.P., Wang K.Q. (2014). Heavy metal ions retention by bi-functionalized lignin: Synthesis, applications, and adsorption mechanisms. J. Ind. Eng. Chem..

[41] Wang L., Wang X., Chen Z.Y., Ma P.C. (2013). Effect of doubly organo-modified vermiculite on the properties of vermiculite/polystyrene nanocomposites. Appl. Clay Sci..

[42] Liu P. (2008). Preparation and characterization of conducting polyaniline/silica nanosheet composites. Curr. Opin. Solid State Mater. Sci..

[43] Zhou S.Y., Xue A.L., Zhao Y.J., Wang Q.W., Chen Y., Li M.S., Xing W.H. (2011). Competitive adsorption of Hg^2+^, Pb^2+^ and Co^2+^ ions on polyacrylamide/attapulgite. Desalination.

[44] Wu R., Liu J., Wu Q. Study on Preparation of Chelating Flocculant and Its Performance of Removing Cadmium. Proceedings of the 2011 International Symposium on Water Resource and Environmental Protection (ISWREP).

[45] Tran L., Wu P.X., Zhu Y.J., Yang L., Zhu N.W. (2015). Highly enhanced adsorption for the removal of Hg(II) from aqueous solution by Mercaptoethylamine/Mercaptopropyltrimethoxysilane functionalized vermiculites. J. Colloid Interface Sci..

[46] Li A., Liu R.F., Wang A.Q. (2005). Preparation of starch-graft-poly(acrylamide)/attapulgite superabsorbent composite. J. Appl. Polym. Sci..

[47] Sing K.S.W., Everett D.H., Haul R.A.W., Moscou L., Pierotti R.A., Rouquerol J., Siemieniewska T. (1985). Reporting physisorption data for gas solid systems with special reference to the determination of surface-area and porosity (Recommendations 1984). Pure Appl. Chem..

[48] Valente J.S., Tzompantzi F., Prince J., Cortez J.G.H., Gomez R. (2009). Adsorption and photocatalytic degradation of phenol and 2,4 dichlorophenoxiacetic acid by Mg-Zn-Al layered double hydroxides. Appl. Catal. B Environ..

[49] Jeon C., Holl W.H. (2003). Chemical modification of chitosan and equilibrium study for mercury ion removal. Water Res..

[50] Huang X., Hou X., Song F., Zhao J., Zhang L. (2016). Facet-Dependent Cr(VI) Adsorption of Hematite Nanocrystals. Environ. Sci. Technol..

[51] Luo P., Zhang J.-S., Zhang B., Wang J.-H., Zhao Y.-F., Liu J.-D. (2011). Preparation and Characterization of Silane Coupling Agent Modified Halloysite for Cr(VI) Removal. Ind. Eng. Chem. Res..

[52] Ye G., Bai F.F., Chen G.J., Wei J.C., Wang J.C., Chen J. (2012). A novel well-ordered mesoporous organosilica specialized for highly selective recognition of Pb(II) by host-guest interactions. J. Mater. Chem..

[53] Nonkumwong J., Ananta S., Srisombat L. (2016). Effective removal of lead(II) from wastewater by amine-functionalized magnesium ferrite nanoparticles. RSC Adv..

[54] Da’na E. (2017). Adsorption of heavy metals on functionalized-mesoporous silica: A review. Microporous Mesoporous Mater..

[55] Lagergren S. (1898). About the Theory of So-Called Adsorption of Soluble Substances Zur Theorieder Sogenannten Ad sorption Geloster Stoffe. Kungliga Svenska Vetenskapsakademiens Handlingar.

[56] Ho Y.S., McKay G. (2000). The kinetics of sorption of divalent metal ions onto sphagnum moss peat. Water Res..

[57] Ho Y.S., McKay G. (1999). Pseudo-second order model for sorption processes. Process Biochem..

[58] Langmuir I. (1916). The constitution and fundamental properties of solids and liquids. Part I. Solids. J. Am. Chem. Soc..

[59] Langmuir I. (1917). The constitution and fundamental properties of solids and liquids. II. Liquids. J. Am. Chem. Soc..

[60] Vadivelan V., Kumar K.V. (2005). Equilibrium, kinetics, mechanism, and process design for the sorption of methylene blue onto rice husk. J. Colloid Interface Sci..

[61] Gong B., Wu P., Huang Z., Li Y., Yang S., Dang Z., Ruan B., Kang C. (2016). Efficient inhibition of heavy metal release from mine tailings against acid rain exposure by triethylenetetramine intercalated montmorillonite (TETA-Mt). J. Hazard. Mater..

[62] Tirtom V.N., Dincer A., Becerik S., Aydemir T., Celik A. (2012). Removal of lead (II) ions from aqueous solution by using crosslinked chitosan-clay beads. Desalination Water Treat..

[63] Jiang M.Q., Wang Q.P., Jin X.Y., Chen Z.L. (2009). Removal of Pb(II) from aqueous solution using modified and unmodified kaolinite clay. J. Hazard. Mater..

[64] Deng Y.H., Gao Z.Q., Liu B.Z., Hu X.B., Wei Z.B., Sun C. (2013). Selective removal of lead from aqueous solutions by ethylenediamine-modified attapulgite. Chem. Eng. J..

[65] Rafiei H.R., Shirvani M., Ogunseitan O.A. (2016). Kinetics and thermodynamics of Pb sorption onto bentonite and poly(acrylic acid)/bentonite hybrid sorbent. Desalination Water Treat..

[66] Liang X.F., Hou W.G., Xu Y.M., Sun G.H., Wang L., Sun Y., Qin X. (2010). Sorption of lead ion by layered double hydroxide intercalated with diethylenetriaminepentaacetic acid. Colloids Surfaces A Physicochem. Eng. Asp..

[67] Datta D., Uslu H. (2017). Adsorptive Separation of Lead (Pb^2+^) from Aqueous Solution Using Tri-n-octylamine Supported Montmorillonite. J. Chem. Eng. Data.

[68] Solener M., Tunali S., Ozcan A.S., Ozcan A., Gedikbey T. (2008). Adsorption characteristics of lead(II) ions onto the clay/poly(methoxyethyl)acrylamide (PMEA) composite from aqueous solutions. Desalination.

